# A Survey of White Matter Neurons at the Gyral Crowns and Sulcal Depths in the Rhesus Monkey

**DOI:** 10.3389/fnana.2017.00069

**Published:** 2017-08-15

**Authors:** Farzad Mortazavi, Samantha E. Romano, Douglas L. Rosene, Kathleen S. Rockland

**Affiliations:** Department of Anatomy and Neurobiology, Boston University School of Medicine Boston, MA, United States

**Keywords:** interstial neurons, nonhuman primate, NeuN, cerebral cortex, subplate

## Abstract

Gyrencephalic brains exhibit deformations of the six neocortical laminae at gyral crowns and sulcal depths, where the deeper layers are, respectively, expanded and compressed. The present study addresses: (1) the degree to which the underlying white matter neurons (WMNs) observe the same changes at gyral crowns and sulcal depths; and (2) whether these changes are consistent or variable across different cortical regions. WMNs were visualized by immunohistochemistry using the pan-neuronal label NeuN, and their density was quantified in eight rhesus monkey brains for four regions; namely, frontal (FR), superior frontal gyrus (SFG), parietal (Par) and temporal (TE). In all four regions, there were about 50% fewer WMNs in the sulcal depth, but there was also distinct variability from region to region. For the gyral crown, we observed an average density per 0.21 mm^2^ of 82 WMNs for the FR, 51 WMNs for SFG, 80 WMNs for Par and 93 WMNs for TE regions. By contrast, for the sulcal depth, the average number of WMNs per 0.21 mm^2^ was 41 for FR, 31 for cingulate sulcus (underlying the SFG), 54 for Par and 63 for TE cortical regions. Since at least some WMNs participate in cortical circuitry, these results raise the possibility of their differential influence on cortical circuitry in the overlying gyral and sulcal locations. The results also point to a possible role of WMNs in the differential vulnerability of gyral vs. sulcal regions in disease processes, and reinforce the increasing awareness of the WMNs as part of a complex, heterogeneous and structured microenvironment.

## Introduction

The structural and connectional heterogeneity of cortical sulcal and gyral regions is well known in the context of laminar deformation (Welker, 1990) and, more recently, in the context of what may be increased axonal fiber density near the gyral crowns (e.g., Nie et al., 2012; Deng et al., 2013). Overlooked in these discussions is what happens to the population of white matter neurons (WMNs or “interstitial neurons”) in the superficial white matter (WM) underlying layer 6. These neurons comprise a phylogenetically conserved mixed population of excitatory and inhibitory neurons, and persist in the adult, with some area- and species-specific variability (discussed in Mortazavi et al., 2016). The functional roles of this heterogeneous population are largely unknown; and are likely to change over time (Friedlander and Torres-Reveron, 2009; Hoerder-Suabedissen and Molnár, 2012). Several studies point to a contribution to cognitive processes, in that the brains of schizophrenic subjects have been reported to have an increased number of WMNs (Akbarian et al., 1996; Connor et al., 2011; Yang et al., 2011). Transcriptomic analyses similarly point to some association with cognitive conditions (Hoerder-Suabedissen et al., 2013; Hoerder-Suabedissen and Molnár, 2015).

From qualitative observations alone, it is obvious that WMNs are more numerous near the gyral crowns than in the sulcal depth. In the present study we addressed the degree to which the population of underlying WMNs observes the same deformations, and whether there are region-specific differences in WMN density in the two locations. Such differences in WMN density could functionally impact circuitry in the overlying cortical gray matter and also impact WM tractography (e.g., Reveley et al., 2015). In addition, such differences could be relevant to the etiology and progression of pathological conditions; that is, both traumatic brain injury (TBI) and Alzheimer’s pathology differ between gryral crowns and sulcal depths with the depths considered as selectively vulnerable (McKee et al., 2013; Arendt et al., 2016).

In a previous survey of superficial and deep WMN distribution in the rhesus monkey (Mortazavi et al., 2016), we reported an average density of about 40 superficial WMNs per 0.16 mm^2^ across frontal (FR), temporal (TE) and parietal (Par) cortical regions. Average WMN density was similar for TE and Par regions, but lower in FR. No significant density differences were observed between a group of four young adult (6.0–7.8 years old) and four older monkeys (20.5–28.7 years old) suggesting that WMNs, like those in the cortical gray matter, are stable across age in the adult (e.g., Peters et al., 1998; Giannaris and Rosene, 2012). In contrast, the population of WMNs (a persisting remnant of the subplate) is subject to dynamic processes and preferential cell death during early development (Kostovic and Rakic, 1980; Chun and Shatz, 1989; Kostović et al., 2011; Judaš et al., 2013).

**Figure 1:**
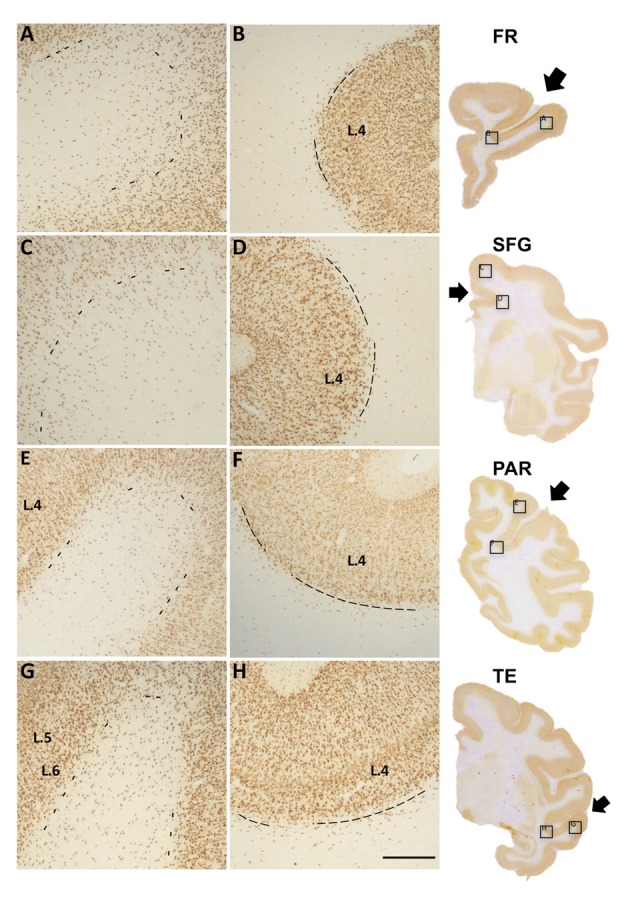
Photomicrographs of Neu-N labeled white matter neurons (WMNs) in the gyral crown **(A,C,E,G)** and corresponding sulcal depth **(B,D,F,H)** in the frontal **(A,B)**, cingulate/superior frontal gyrus (SFG) **(C,D)**, parietal **(E,F)** and temporal regions **(G,H)**. The border between layer 6 and the subjacent WM is indicated by dashed lines. Coronally sectioned histological sections (at right) indicate approximate AP level and location of the gryal and sulcal photomicrographs (in boxed locations). Short arrows, from top to bottom, point to principal sulcus, cingulate sulcus, intraparietal sulcus (IPS) and superior temporal sulcus (STS). L = layer, Scale bar = 500 μm **(A–H)**.

The present investigation compares WMN density specifically in the gyral crowns and sulcal depths of the same eight monkeys (Figure 1). Here, we have adopted a slightly larger sampling frame (region of interest, ROI; 0.21 mm^2^), as better conforming to sulcal and gyral topography. In order to achieve wider representation of association areas, we added a comparison between the cingulate sulcus and the overlying superior frontal gyrus (SFG). In brief, we found that for all four cortical regions, the sulcal density of WMNs was about 50% that at the gyral crown; but in both gyral and sulcal locations, WMN density was least for the SFG region and greatest for the TE.

## Materials and Methods

### Subjects and Tissue Preparation

The present investigation utilized tissue from the eight male rhesus monkeys (*Macaca mulatta*) that were the basis for our previous study (Mortazavi et al., 2016). Briefly, all animals were obtained from national primate centers and had known birth dates and health records which were screened to ensure that they were free from any history of disease or experimentation that might compromise the brain. All were part of other ongoing studies and were housed at the Laboratory Animal Science Center on the Boston University Medical Campus (BUMC). This facility is managed by a licensed veterinarian, staffed by trained and accredited laboratory animal staff and fully accredited by the Association for the Assessment and Accreditation of the Laboratory Animal Care. All procedures conformed to the National Institutes of Health guidelines and the Institute of Laboratory Animal Resources Commission on Life Sciences’ *Guide for the care and use of laboratory animals* (1996). All procedures were approved by the BUMC Institutional Animal Care and Use Committee.

After all behavioral testing and other procedures were completed, subjects were tranquilized with ketamine (10 mg/kg, intramuscular), deeply anesthetized with sodium pentobarbital (15 mg/kg, intravenous to effect), and euthanized by exsanguination during transcardial perfusion with 4% Krebs buffer at 4°C, followed by 4% paraformaldehyde in phosphate buffer (0.1 M, pH 7.4) at 37°C. Brains were blocked *in situ*, in the coronal plane just posterior to the splenium, removed from the skull, weighed, post-fixed overnight in 4% paraformaldehyde for no more than 18 h, and transferred to cryoprotectant solution to eliminate freezing artifact (Rosene et al., 1986). Blocks were flash frozen at −75°C and stored at −80°C until sectioned at 30 μm thickness into 10 repeating series of sections. Series not processed immediately were stored in cryoprotectant (15% buffered glycerol) at −80°C until removed, thawed and batched processed for immunohistochemistry (Estrada et al., 2017).

### Immunohistochemistry

As described more fully in Mortazavi et al. (2016), sections were thawed, and rinsed three times for 5 min in 0.05 M Tris-buffered saline (TBS; pH 7.4) to remove the cryoprotectant. To quench endogenous peroxidases, sections were incubated for 30 min in 0.05 M TBS and 1% hydrogen peroxide. After three 5 min washes in 0.05 M TBS, sections were incubated for 1 h in a blocking solution of 10% Normal Goat Serum (NGS) and 0.4% Triton-X in 0.05 M TBS before incubation for 48 h at 4°C with gentle agitation in mouse anti-NeuN IgG (1:10,000; MAB377, Chemicon, Temecula, CA, USA), in 0.05 M TBS containing 2% NGS and 0.1% Triton-X. Following this incubation, the sections were again washed, and further processed by a 2 h incubation period with the secondary antibody (goat anti-mouse, 1:600; Vector, Burlingame, CA, USA), in 0.05 M TBS with 2% NGS, and 0.4% Triton-X. After washing, sections were incubated with an avidin biotinylated horseradish peroxidase complex (ABC, Vector Labs, Burlingame, CA, USA) for 1 h. After another wash cycle, sections were incubated for 7 min in sodium acetate containing 0.55 mM 3-3′-diaminobenzidine (DAB; Sigma, St. Louis, MO, USA) and 0.01% H_2_O_2_. Sections were washed, mounted onto gelatin-coated slides, air dried and cover-slipped with Permount mounting medium (ThermoFisher Scientific, Waltham, MA, USA).

### ROIs in this Study

As an extension of Mortazavi et al. (2016), this study focused on four WM regions (SFG, FR, Par and TE). We felt this provided a base comparison of associational cortical areas, from dorsal and ventral streams, and representative of anterior-posterior levels, and with different gray matter cytoarchitecture and connectivity. Analysis of additonal regions was not undertaken because of lack of tissue (for occipital) and, at this time, lack of resources to undertake a more comprehensive study between primary and association WM areas or further detailed subregions.

The nomenclature for gross anatomical, macro WM subdivisions is much less well established than that for the overlying gray matter or for defined axon tracts within the WM. For gyral WM regions, in particular, nomenclature sometimes adheres to gross anatomical distinctions (“superior temporal gyrus, STG”) but other times follows gray matter architectonic subdivisions (“area TEd”). In this study, we used “frontal region” to denote the territory from the anterior tip of the principal sulcus to the arcuate spur; the “parietal region”, the zone from the anterior tip of the intraparietal sulcus (IPS) to the posterior part of the superior temporal sulcus (STS); and the “temporal region”, from the mid-amygdala to the anterior IPS. For the temporal region, counts were subdivided so as to correspond to the STG and the gyrus ventral to the STS (TEd; Figure 1).

The identification of the cingulate sulcus and its nomenclature are straightforward. The dorsally adjoining gyrus, however, which we used for gyral counts in this region, was more problematic. This territory, from the spur of the arcuate sulcus posterior to the central sulcus, underlies several architectonic cortical regions, including the supplementary motor, and somatomotor functional cortical domains. In the human brain, it would correspond to the gross anatomical “SFG”, and we adopted this term for the sake of convenience.

### Estimation of WMN Density in Sulcal and Gyral Locations

The results in the current study are based on the same tissue as used in Mortazavi et al. (2016). ROIs were digitized at on a Zeiss Axiophot microscope using a 5× objective. These high resolution images were then stitched using the pair-wise stitch function in Fiji (Schindelin et al., 2012), and further zoomed for counting purposes, to a total magnification of 120–150×. Two to four sections per animal, per region generated a total of 147 counting frames in the sulcus and 125 in the gyrus (per region: 57 in SFG, 72 in FR, 75 in Par and 68 in TE).

**Figure 2:**
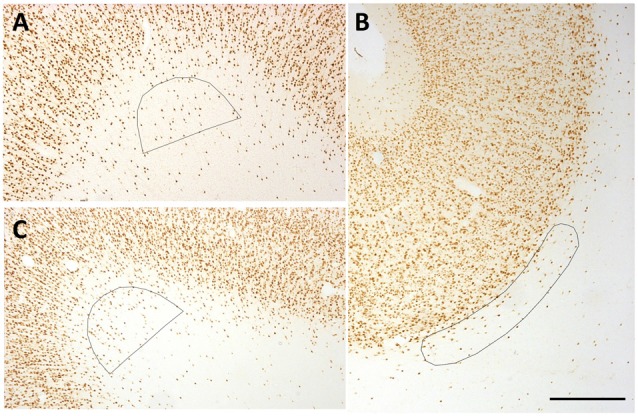
Representative counting frames (0.21 mm^2^). **(A,C)** Semi-circles delineate counting zones at SFG **(A)** and temporal region (TE) **(C)** gryal crowns. **(B)** Ovoid outline delineates counting zone for a Par sulcal region. Scale bar = 500 μm.

Polygons of area 0.21 mm^2^ were created with the Freehand selection tool in ImageJ (NIH, Bethesda, MD, USA). Polygons approximated a semi-circle or curved cone for gyral crowns, and crescent for sulcal depths (Figure 2). Neuron counts were carried out by using the cell counter tool in ImageJ and entered into Excel sheets. Counts were carried out by one investigator (AM), and partially replicated by two or three other scorers (AK, LG, SR, or KSR), with a quality control criterion that counts differed by less than 10%. As in Mortazavi et al. (2016), all neurons within the bounded ROI were counted. As noted in other quantitative studies of WMNs (e.g., Yang et al., 2011), designed-based stereological counting is not easily applied to the relatively borderless WM expanse.

Density measurements were made from four association areas; namely, FR, SFG, Par and TE. These represented a broad sample spanning the length of the cerebral hemisphere and areas with different gray matter cytoarchitecture and connectivity. Given the functional importance of the TE region, we further distinguished two subdivisions in this region, corresponding to the STG, between the lateral and superior temporal sulci, and TEd (ventral to the STS; Saleem and Logothetis, 2007). The border of layer 6 and WM was judged by a fall-off in neuron density and a frequent change in preferred cellular orientation, from perpendicular to parallel to the overlying pia surface. To minimize the risk of including the deepest portions of layer 6, we systematically placed the superficial border of the ROI slightly beneath the qualitatively judged deep boundary of layer 6 (see Figure 2).

To evaluate changes in WMN density proceeding away from the gryral crown, in the two TE subdivisions, we used a counting frame in the shape of a narrow rectangle (0.2 mm × 5.0 mm). One edge of this rectangular counting frame was placed at the gyral crown, and four underlying zones (each 1.25 mm) were demarcated medially toward the sulcal depth. The rectangle was placed approximately in the middle of the gyral core, so as to avoid possible overlap with domains associated with gyral walls.

### Statistical Analysis

In our previous investigation, we found no change in WMN numbers between young adult and aged monkeys (Mortazavi et al., 2016). Hence in the current study, we pooled the old and young groups for all analyses. In contrast to the earlier study, the combined data from the larger sample were distributed normally as determined by a Shapiro Wilk test of normality, enabling the use of parametric statistics in the current study.

Statistical analysis was carried out using a within subjects design, and Bonferroni adjustments were made for multiple comparisons. To determine whether density of WMNs was different in the gyral crown vs. the sulcal depth, a one-way within ANOVA was used. To determine regional differences in the density of WMNs, a two-way repeated measures within-subjects ANOVA was used (two locations: gyrus, sulcus × four regions: FR, SFG, Par and TE). To determine if there was a statistically significant change in the WMN density in moving from the gyral crown towards deep WM, a repeated-measures ANOVA was used. All data are reported as mean ± SEM, and alpha was set at 0.05.

## Results

### Density of WMNs in Gyral Crown vs. Sulcal Depth

**Figure 3:**
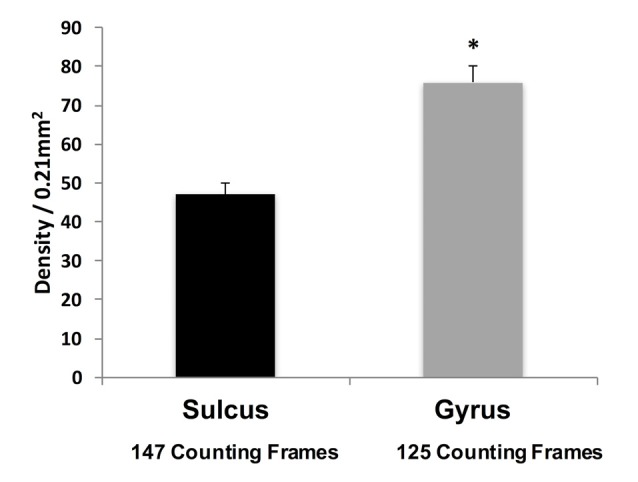
Density of WMNs in sulcal depth and gyral crown in eight monkeys, averaged across all four regions. *p < 0.05, gyrus compared to sulcus.

Qualitative observations suggest that WMNs are distinctly more numerous in the gyral crowns than sulcal depths (Figure 3). To confirm the apparent difference, the density of WMNs in the gyral crown and sulcal depths of our four cortical regions (FR, SFG, Par and TE) was quantified. Analysis showed an average of 1.5× more WMNs in gyral crowns (75 ± 4 WMNs per 0.21 mm^2^) than in sulcal depths (about 47 ± 3 WMNs per 0.21 mm^2^; *F*_(1,7)_ = 58.41, *p* < 0.05).

### Density of Gyral and Sulcal WMNs Across Cortical Regions

**Figure 4:**
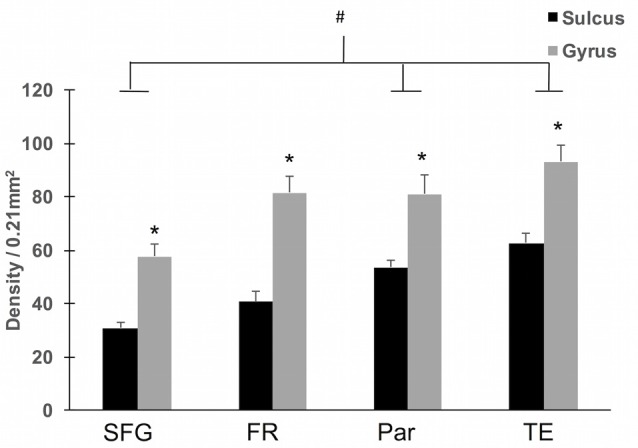
Regional density of WMNs in sulcal depths and gyral crowns. All four regions showed significantly greater WMN density in the gyral crown compared to the sulcal depth (*p < 0.05). Across the four regions, both sulcal and gyral WMN density was least for cingulate (Cing)/SFG and greatest for the TE region. Temporal and parietal (Par) regional counts were significantly greater than counts for Cing/SFG (^#^*p* < 0.05).

**Figure 5:**
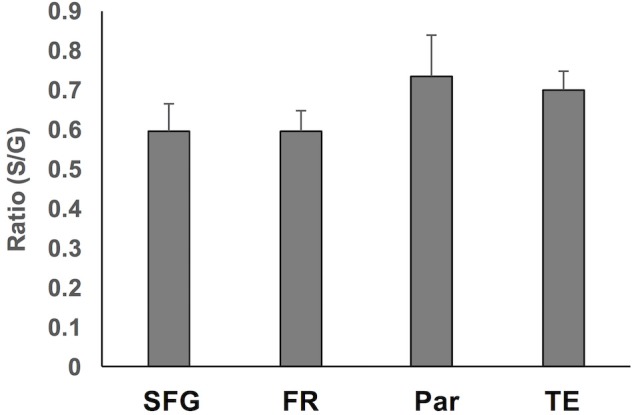
Summary of the ratio of sulcal to gyral WMN counts across the four regions. These were not significantly different.

To assess region-specific differences in density of WMNs, the two-way ANOVA indicated that the global average difference summarized in Figure 3 is maintained at the region-specific level (Figure 4); and in all four regions, gyral WMN density was greater than that in the sulcus (*F*_(1,12)_ = 24.93, *p* < 0.05). For gyral locations, WMN density ranged from a low of 57 ± 4 neurons per counting frame in the SFG region to 93 ± 6 WMNs per counting frame in TE (TEd = 95 ± 5; STG = 81 ± 5). For FR and Par, we found 81 ± 5 and 80 ± 6 WMNs per counting frame, respectively. For sulcal locations, WMN density showed the same trend; that is, least for the SFG/cingulate region (31 ± 4 WMNs per counting frame), intermediate for FR (41 ± 3 WMNs) and Par (54 ± 2 WMNs), and highest for TE (63 ± 3 WMNs; TEd = 58 ± 4, STG = 67 ± 4). Additionally, *post hoc* analysis showed that there are significantly more WMNs in both TE and Par (both sulcal and gyral) than in FR. While the ratio of sulcal/gyral WMNs varied in the different regions (FR (0.59), SFG (0.59), Par (0.73) and TE (0.70)), the differences were not statistically significant (Figure 5).

### Density of WMNs Along Gyral Depth

**Figure 6:**
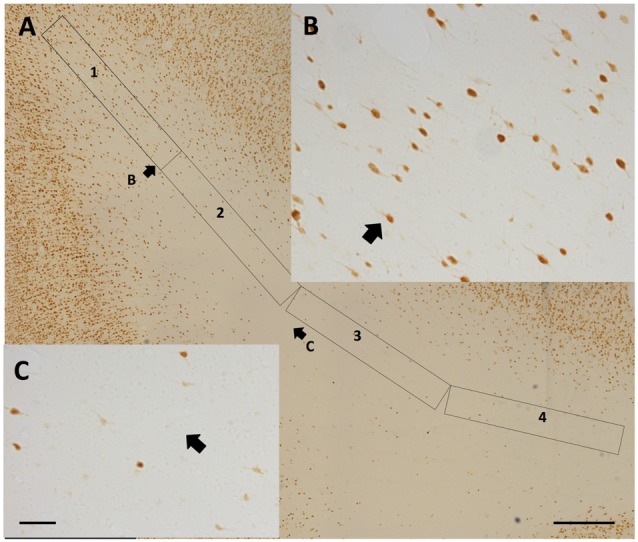
**(A)** Representative 4-zone (1.125 mm long) rectangular counting frame, for determining WMN density as a function of distance from the gyral crown (at upper left). Zones 3 and 4 are tilted slightly, in order to remain near the middle of the gyrus. **(B)** Higher magnification from the distal portion of zone 1. **(C)** Higher magnification from the proximal portion of zone 3. Short arrows in **(A,B)** and **(A,C)** indicate corresponding fields. Scale bars = 500 μm in **(A)**; 50 μm in **(B,C)**.

**Figure 7:**
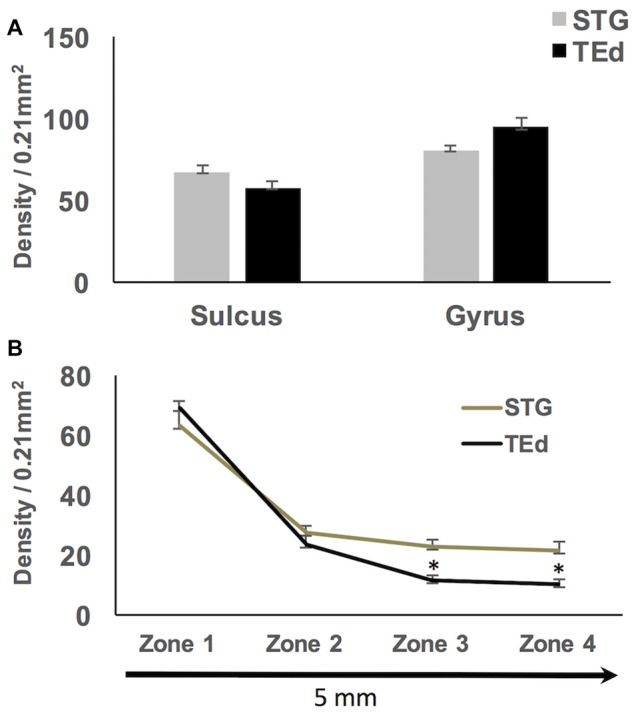
**(A)** No significant differences in density of sulcal and gyral WMNs were found between superior temporal gyrus (STG) and inferior temporal gyrus (TEd). **(B)** Significantly progressive fall-off in WMN density with distance from the gyral crown; the fall off in density of WMNs was significantly different between STG and TEd at the most distal zones. *p < 0.05.

Qualitative observations of all four regions suggest that WMNs become progressively sparser with distance from the gyral crown (Figure 6). As a first step, we compared the density of WMNs individually in STG and TEd. Both regions had fewer WMNs in the sulcal location. The sulcal/gyral proportions were slightly different, with a ratio of 83% for STG and 60% for TEd, though this was not statistically significant (Figure 7A). For the two temporal subregions, we quantified WMN density from the gyral crown toward the sulcal depth by tabulating WMN number along a rectangular counting frame (0.2 mm wide by 5.0 mm in length), divided into four adjacent segments or zones (0.2 mm × 1.25 mm each in; Figure 6A). As summarized in Figure 7, we found for zone 1, immediately near the gyral crown, 70 ± 2 WMNs in TEd and 63 ± 4 in STG. We also found a consistent reduction from zones 1 and 2, where comparable numbers in zone 2 are, for TEd, 26 ± 3 neurons and, for STG, 28 ± 3 (per 0.25 mm^2^). There is a small reduction from zone 2 to 3 but otherwise numbers are stable from zone 3 to 4; namely, for zone 3, 12 ± 1 neurons in TEd and 22 ± 2 in STG; and for zone 4, 10 ± 1 in TEd and average 21 ± 3 in STG. Statistical analysis confirmed that the drop in WMN density was significant in both STG and TEd, proceeding from the gyral crown medially, towards deep WM (*F*_(3,16)_ = 98.464, *p* < 0.05). Further, *post hoc* analysis showed that there are significantly more WMNs in zones 3 and 4 of STG as compared to TEd (Figure 7B).

Individual tissue sections show a less regular pattern, in that the number of WMNs can remain constant over zones 2–4, or the number in zone 4 can even be slightly higher, as assessed within our rectangular counting frame. This is at least partially explained by an irregular pattern in the arrangement of WMNs.

### Pattern of Cell-Free Gaps

**Figure 8:**
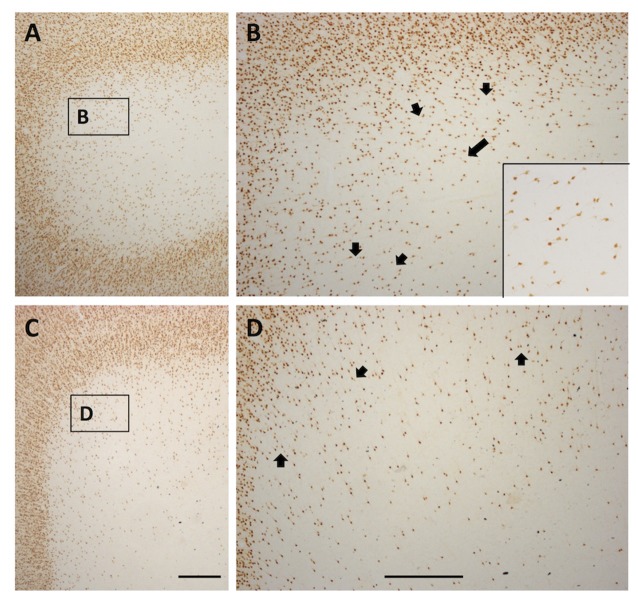
Photomicrographs from a TE (**A** and higher magnification **B**) and SFG gyral region (**C** and higher magnification **D**). Both show a quasi-circular pattern, with scattered WMNs around the perimeter of cell-free gaps. Short arrows in **(B,D)** indicate several of the circular, cell-free gaps. Long arrow in** (B)** points to the boxed region shown at higher magnification in the inset at lower right. Boxes in **(A,C)** correspond to higher magnification views in **(B,D)**. In **(A)**, lateral is to the left, and in **(C)**, medial is to the left. Scale bars = 500 μm.

In gyral locations, the arrangement of WMNs is not uniform, but rather exhibits distinct, usually circular neuron-free zones (Figure 8). These were relatively common, and were observed in all four regions. Circular zones ranged from 120 μm to 250 μm in diameter. Quantification of size differences will require further investigation in strict serial sections or in thick tissue slabs.

## Discussion

In this study, we first determined the relative density of WMNs in sulcal and gyral locations and assessed variability across four regions of association cortex. In our material, for both gyral crown and sulcal depth locations, the temporal region had the highest WMN density, followed by parietal and frontal regions, with the cingulate and SFG region having the lowest (Figure 4). This is generally consistent with the overall averages reported in our previous study. That study did not distinguish between the gyral crest and depth (Mortazavi et al., 2016), but did show an overall lower density of superficial WMNs in frontal cortex, in comparison with temporal and parietal regions. An earlier investigation of the subpopulation of WMNs positive for NADPH-diaphorase reported area specific differences, where the number of WMNs (under 1.0 mm^2^ in six cortical areas, Table 1 in Barone and Kennedy, 2000) was greatest in area 6, intermediate in temporal areas, and least for occipital (Figure 3 in Barone and Kennedy, 2000). In contrast, studies of human postmortem cortex report WMN density as greater for frontal cortex, followed by cingulate cortex, with visual and temporal cortices having the lowest density (García-Marin et al., 2010; and see “Discussion” Section in Mortazavi et al., 2016).

Second, we determined the apparent fall-off of WMN density as a function of distance from the gyral crown, focusing on the superior and middle temporal gyri (STG and TEd). For these two temporal subregions, zone 1 (1.125 mm subjacent to the crown) contained the most WMNs, and zone 2 (the next 1.125 mm) consistently had fewer neurons. While there was overall a progressive fall-off in density across the four zones (of about 50% from zone 1 to zone 4), there were also individual sections where zones 3 or 4 showed a localized increase, or where zones 2–4 were relatively uniform. In the distal portion of the gyrus (furthest from the pia), the density of gyral WMNs is close to the average density of WMNs in the deep WM (10–12 WMNs per 0.21 mm^2^, as adjusted from 0.16 mm^2^ in Mortazavi et al., 2016).

There have been relatively few quantitative data on fall-off of WMNs along a gyral depth; but in one anatomical study of patients with focal epilepsy (Loup et al., 2009), density of WMNs was assessed along an 8.0 mm distance deep from the gyral crown in temporal cortex. In the immediate vicinity of layer 6, beneath the gyral crown, about 60 neurons were scored per 0.98 mm^2^. This approximates our zone 1, where we scored (per 0.25 mm^2^), 39–84 neurons or 38–94 neurons, respectively in STG and TEd. Loup et al. (2009; Figure 3) further report a progressive decline over the next 0.25 mm (to 35 WMNs) and 0.75 mm (to 25 WMNs) before there is a leveling off, at 5–10 neurons for the remaining 6.0 mm. By comparison, our zone 2 (1.125 mm deep to zone 1) averaged about 25 WMNs, and the distal zone 4 (3.75–5.00 mm deep to the gyral crest) averaged between 10 and 25 WMNs (see Figure 7). More work on the issue of intra-gyral organization would be useful, in terms of a larger sample, more cortical regions, and finer analysis by GABAergic and glutamatergic subtypes. Receptor profiles, for m^2^ muscarinic receptors (Smiley et al., 1998) or GABA_A_ receptor subtypes (Loup et al., 2009), complexin 3 and other markers (Table 2 in Kanold and Luhmann, 2010) are already available for finer dissections.

Third, we report that there is an inhomogeneous arrangement of WMNs, especially in the outer 2.0 mm (our zones 1 and 2) nearest the gyral crown, where WMNs typically exhibit a pattern of quasi-regular neuron-sparse circles, 120–250 μm in diameter. Similar neuron-free microzones have been reported in human cortical tissue (Loup et al., 2009; their Figure 7A) but have been postulated to relate to the epileptic processes or pathologies. Cell-free circular zones can be documented in the figures of several other studies (Delalle et al., 1997, Figure 1 for NPY neurons in middle frontal gyrus of humans; Figure 6 in Smiley et al., 1998, for WMNs neurons positive for m2 receptor; García-Marin et al., 2010, Figure 2 for Neu-N positive WMNs in human). Possibly, this quasi-circular pattern may have an orderly relationship to axonal bundles, vasculature, or glia compartments. This could be investigated by double immunohistochemistry and quantitative analysis in serial sections of in small tissue slabs.

### Gyral and Sulcal Heterogeneity of WMNs: Circuitry and Gene Expression

At least some, if not all, WMNs are connectionally integrated into gray matter circuitry (Clancy et al., 2001; Tomioka and Rockland, 2007; Suárez-Solá et al., 2009; von Engelhardt et al., 2011). Thus, the differential density of WMNs at the gyral and sulcal locations can be taken to imply a differential influence of these neurons in relationship to the corresponding gyral and sulcal cortical gray matter. There could be different numbers of synapses, different postsynaptic targets, or different axonal collateralization patterns at gyral crowns and sulcal depths. For example, retrograde tracing experiments demonstrate that many WMNs at the gyral crest project corticothalamically (for monkey frontal cortex: Figures 10, 12 in Giguere and Goldman-Rakic, 1988). The smaller number of WMNs in the sulcal depth may mean that there are fewer corticothalamic projections from sulcal locations. Further, if corticothalamic WMNs share features of the overlying layer 6 corticothalamic (type 1) neurons (Rockland, 1996), these potentially would have collaterals to the reticular nucleus of the thalamus, the thalamus and the overlying cortical gray matter (in layer 4). Interestingly, recent studies in mouse report that the subpopulation of complexin-3 positive subplate neurons projects both to cortical thalamorecipient layers and to the thalamus, based on immunohistochemical localization of the presynaptic terminals (Hoerder-Suabedissen et al., 2016; Viswanathan et al., 2016).

A small population of WMNs, seemingly in a gyral location, contributes to long distance cortico-cortical projections (Mohedano-Moriano et al., 2015 and (identified as GABAergic), Tomioka and Rockland, 2007).

Sulcal and gyral specialization (“folding”) has been actively discussed in the context of cortical development (see Lewitus et al., 2013; Zilles et al., 2013; Sun and Hevner, 2014; Striedter et al., 2015). Recent evidence suggests the location of folds and fissures (i.e., gyral crown and sulcal depth) is mirrored by regional variation in progenitor cell proliferation (de Juan Romero et al., 2015); and comparison of gyral and sulcal progenitor transcriptomes demonstrates “thousands of genes” differentially expressed in prospective folds (gyri) or fissures (sulci; reviewed in Fernández et al., 2016). In brains of young ferrets (P6), particular combinations of genes that are modularly expressed in the outer subventricular zone correspond to the location of folds and fissures, and the combinations are thought to differ depending on the specific gyrus or sulcus (Fernández et al., 2016). If WMNs have the same expression differential, this would suggest that they develop in concert with overlying cortical neurons but if they have a different pattern it would raise the possibility that they constitute a unique functional entity. Since, moreover, WMNs occur in lissencephalic brains, it seems more likely that any contribution to cortical folding is contributory rather than key.

### Gyral and Sulcal Heterogeneity: In Disease

An increased density of WMNs has consistently been associated with schizophrenia (Eastwood and Harrison, 2003; Connor et al., 2011; Kostović et al., 2011; Yang et al., 2011; Joshi et al., 2012), and there are reports of an increased frontal density of WMNs in fetal alcohol syndrome in macaque (Burke et al., 2009). In these conditions, less information is available about changes in the density of WMNs in relation to gyral/sulcal locations; but one postmortem investigation of epileptic brains reports an overall increased density of WMNs (Figure 3 in Loup et al., 2009). Age-related and Alzheimer’s-related changes occur in the cholinergic system, and these may differentially effect gyral, intra-gyral, and sulcal regions (Mesulam, 2013). Both plaques and tangles are reported as more prominent in sulcal depths than gyral crowns (Arendt et al., 2016). A common discussion point has been that superficial WM is particularly vulnerable due to a unique environment of late-myelinating U-fibers and late-myelinating oligodendrocytes (Haroutunian et al., 2014; Phillips et al., 2016a,b). The lower density of WMNs in the sulcal depths may be a contributing factor if, for example, these serve a protective role via neurovascular or synaptic processes (Rockland and Nayyar, 2012).

### Gyral and Sulcal Heterogeneity: In Imaging

Finally, as observed in Reveley et al. (2015), the differential neuropil environment of sulci and gyri, which includes the dendritic and axonal meshwork of WMNs, impacts on the accuracy of tracking algorithms in DTI and fMRI protocols. In the *in vivo* imaging field, the segmentation of gray and WM, and in particular the accurate determination of gray matter/WM borders can have major consequences; for example, MR studies commonly assess thickness of cortical gray matter and WM in disease and normal development and aging. Currently, there is no one algorithm acknowledged as the most accurate because segmentation in MR is largely limited by partial volume effects (volume averaging of voxels; Yeh and Tseng, 2013; Yeh et al., 2013; Tohka, 2014; Rullmann et al., 2016). The microstructural organization of WMNs, across individual sulcal and gyral locations, underlines the continued need for better algorithms that are more sensitive to the complexities and ambiguities of anatomical segmentation.

## Conclusion

The density of WMNs at gyral crowns is about 50% greater than at sulcal depths, but there is regional variation. This result raises the possibility that WMNs may have a differential influence on the circuitry and disease vulnerability of the overlying cortex at these specialized locations. Our results reinforce the fact that the subcortical WM is not homogeneous, but rather is a complex microenvironment, consisting of differential arrangements of WMNs and, presumably, glia, among the WM bundles.

## Author Contributions

FM and KSR wrote the manuscript, designed and conducted immunohistochemical experiments, analyzed the data and prepared illustrations. SER contributed to cell counts, digitized the images and assisted with data analysis and figure preparation. DLR guided the overall experimental design and participated in manuscript preparation.

## Conflict of Interest Statement

The authors declare that the research was conducted in the absence of any commercial or financial relationships that could be construed as a potential conflict of interest.

## Abbreviations

Cing: cingulate
FR: frontal region, from the anterior tip of the principal sulcus to the arcuate spur
MRI: magnetic resonance imaging
Par: parietal region, from the anterior tip of the intraparietal sulcus (IPS) to the posterior part of the superior temporal sulcus
SFG: superior frontal gyrus
STG: superior temporal gyrus, between the lateral and superior temporal sulci (STS)
TBI: traumatic brain injury
TE: temporal region (STG + TEd)
TEd: inferior temporal gyrus, just ventral to the superior temporal sulcus
WMNs: white matter neurons.
